# Symptomatic synovial herniation pit—MRI appearances pre and post treatment

**DOI:** 10.1259/bjrcr.20160103

**Published:** 2017-01-05

**Authors:** Liam Kavanagh, Caoimhe Byrne, Eoin Kavanagh, Stephen Eustace

**Affiliations:** Department of Radiology, Cappagh National Orthopaedic Hospital of Ireland, Finglas, Ireland

## Abstract

Herniation pits are small benign lucent oval lesions within the anterior aspect of the superolateral femoral neck and were first described in 1982 by Michael J. Pitt. They are widely believed to occur as a result of mechanical forces from the overlying capsule resulting in herniation of soft tissues and synovium through a small bony defect. More recently, there has been evidence to suggest that femeroacetabular impingement may have a role in their aetiology. We present a case of a 59 -year -old male patient who developed hip pain following a jump from a wall. MRI was performed following failure of conservative management and demonstrated a small herniation pit with surrounding bone oedema. Following flouroscopic intra-articular steroid injection there was complete resolution of the patient’s symptoms and the bone oedema surrounding the herniation pit. We review the potential causes, imaging appearances and potential treatment of synovial herniation pits with an emphasis on the role of radiologically guided intra-articular steroid injection.

## Background

Herniation pits or “Pitt’s pits” were first described by Michael J. Pitt in 1982. On anteroposterior radiographs they can be seen as a small well-delineated lucent oval or sometimes lobulated area within the proximal superior quadrant of the femoral neck approximately 1 cm below the superior femoral neck margin. They have a thin sclerotic rim and represent benign bone pits that are felt to be a result of mechanical forces causing herniation of soft tissue and synovium through a defect in the bone. They are usually a unilateral incidental finding and rarely present with hip pain. Incidence in the general population is said to be 5%,^1^ and they are seen more often in older male patients than in female patients.^2^ More recently, an association with femeroacetabular impingement has been suggested as a possible causative factor of these lesions, which have since also been referred to as juxtacortical fibrocystic changes.^3^ We present the MR imaging appearances of a patient with a symptomatic herniation pit pre and post treatment and in so doing emphasize their potential role as a cause of hip pain, and the associated benefits of treatment with image-guided intra-articular steroid.

## Case report

**Figure 1.**
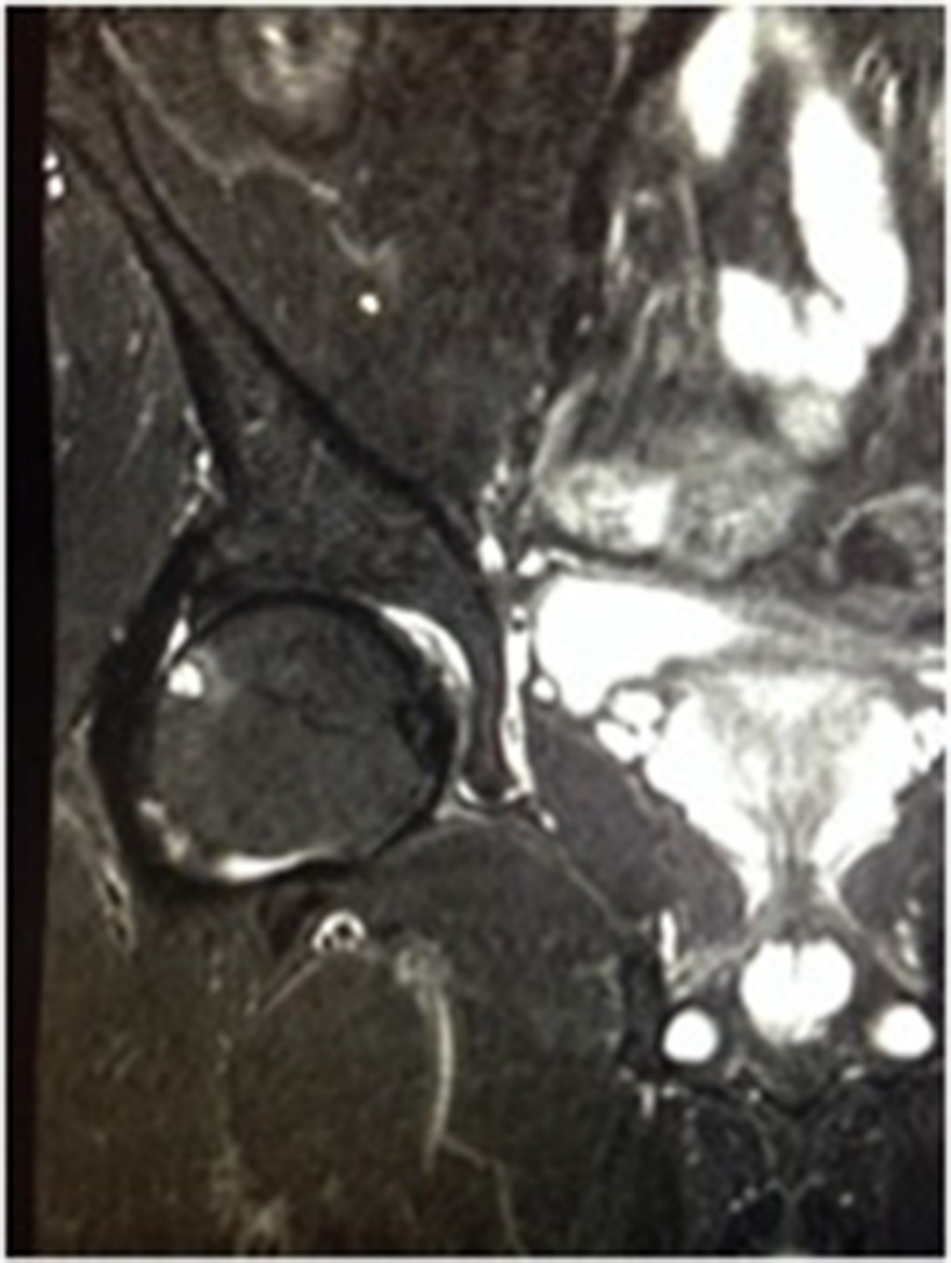
Coronal short tau inversion-recovery image of the right hip demonstrating a 5-mm herniation pit at the anterior aspect of the superolateral femoral head–neck junction with mild surrounding bone oedema.

Mr JA, a 59-year-old active doctor with no significant medical history, presented with a 4-month history of right-sided hip pain radiating to the groin following minor trauma incurred by a jump from a wall. Conservative measures such as rest, non-steroidal anti-inflammatory medication and physiotherapy provided minor relief but failed to resolve the pain. Clinical examination suggested impingement and a labral tear as the source of the pain. The patient went on to have an MRI of the hip, which demonstrated a small synovial herniation pit within the anterior aspect of the superolateral femoral head and neck junction measuring 5 mm with a mild amount of surrounding bone oedema and subtle lateral femoral head–neck junction contour abnormality suggestive of early cam-type impingement morphology. There was no fracture evident and the articular surfaces, labrum and the remainder of the pelvis were normal (Figure 1).

Flouroscopically guided intra-articular steroid injection of 8 mg dexamethasone mixed with 2 cc of 0.25% bupivacaine was performed resulting in symptom improvement immediately following the procedure. Within 6 days the symptoms had completely resolved.

**Figure 2.**
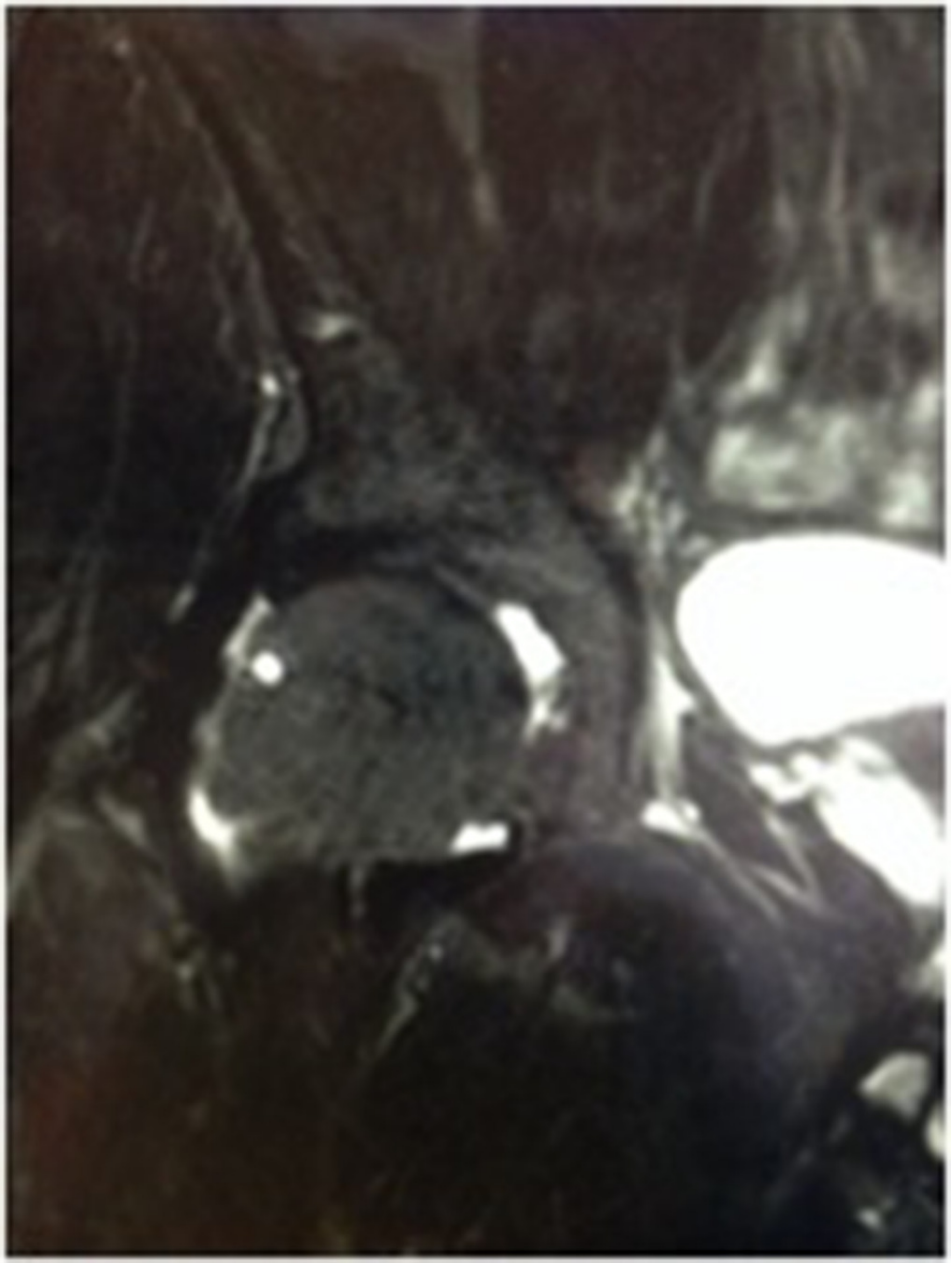
Coronal STIR image of the right hip 1 month post flouroscopic intra-articular steroid injection demonstrating a 5 mm herniation pit in the anterior aspect of the superolateral femoral head–neck junction with resolution of surrounding bone oedema.

Follow-up MRI 1 month later demonstrated complete resolution of the bone oedema surrounding the herniation pit (Figure 2). At 1-year follow-up the patient remained asymptomatic.

## Discussion

In 1882, Allen^4^ described a bony depression in the superolateral quadrant of the femoral neck or “the cervical fossa of Allen.” Young adults and children can demonstrate a small depression in this region; however, the majority of adults will demonstrate a plaque-like bony elevation surrounding and invading into the fossa of Allen.^5^ Histologically this region comprises dense, collagenous and hypocellular tissue overlying neocartilage with reactive new bone underneath.^1^ This area was subsequently termed the “reaction area” by Angel^6^ in 1964 when he discovered 83% of males and 74% of female cadavers to have such surface changes. It was subsequent to this that Pitt described a cavity, which occasionally developed beneath the reaction area via herniation of synovium and soft tissues through perforations or defects in the reaction area, via the mechanical effects of the overlying hip capsule. Histologically, these pits contained fibrocartilage and hyaline tissue walls that were filled with fibroareolar tissue.^1^

The proposed reaction area is felt to be secondary to the mechanical effects of the overlying hip capsule.^7^ As the zona obicularis and iliofemoral ligament converge above the reaction area the capsule becomes thickened and this is felt to contribute to the development of the reaction area, which is supported by Angel’s cadaveric study.^6^ Repetitive flexion and extension of the hip and the subsequent mechanical friction of the iliofemoral ligament laterally, the overlying capsule and the iliopsoas tendon medially likely lead to herniation of soft tissue into defects within the reaction area resulting in the herniation pit.^8^

Generally, these pits remain stable in size; however, they have been seen to enlarge in athletes over time, supporting the theory that mechanical stress and in particular hyperextension of the hip may lead to development of these pits.^9^

More recently, femeroacetabular impingement (FAI) has been suggested as the cause of these herniation pits. FAI occurs when morphological alterations in the femoral head and neck and/or the acetabulum result in contusional lesions to the labrum and cartilage following hip flexion and internal rotation. This is felt to be a causative factor in hip osteoarthritis.^10^ A study by Leunig M et al in 2005 proposed that the cystic changes seen in the superolateral quadrant of the femoral neck were secondary to repetitive mechanical contact of the acetabulum and the femoral neck and not just an incidental finding. They retrospectively compared radiographs of 117 hips with FAI to a control group of 132 hips with developmental dysplasia (DD). They found fibrocystic changes in 33% of the FAI group and in none of the DD group. Furthermore, in 24 patients who underwent joint preserving surgery for FAI they discovered a close spatial relationship between fibrocystic changes noted during the surgery and during dynamic MR arthrography with the hip flexed. They suggest that these fibrocystic changes are a result of impingement rather than due to herniation of synovium. They felt this was further supported by the fact that none of the DD group, who are subject to even more mechanical abrasive effects from the overlying capsule due to the femoral head migrating anterolaterally, had fibrocystic changes, thereby dispelling the belief that these cystic lesions are due to herniation of synovium. Histology of the cystic lesions was examined in seven patients and were found to contain proliferative fibroblasts such as those seen in subchondral ganglia.^3^

With conventional radiography, the pits are shown typically as a small oval or round radiolucency usually with a thin sclerotic rim, less than 1 cm in diameter (but can be seen up to 15 mm), within the anterior aspect of the superolateral quadrant of the femoral neck.

CT shows the pit as a well-defined subcortical lytic pit usually with sclerotic margins and an overlying linear cortical defect. Occasionally, CT will demonstrate a pit that is not visualized on plain radiographs.^11^ MRI typically demonstrates a *T*_1_ low signal, well-defined lesion that is bright on *T*_2_ with a well-defined low signal periphery.

Isotope bone scan will rarely show any increased uptake; however, if there is uptake, it may be explained by stress, resorption or remodelling associated with the formation and natural history of the pit.^12^

The differential considerations include osteoid osteoma, intraosseous ganglion, atypical metastasis and chronic abscess; however, the clinical picture and characteristic imaging findings will usually discount these with reasonable certainty.

The exact aetiology of these characteristic cystic lesions is debated; however, the most recent evidence from Leunig certainly favours impingement morphology as a causative factor.

Bone marrow oedema is a result of increased intravascular pressure and local changes in the capillary wall with subsequent capillary leakage. When located next to a joint it is frequently associated with pain.^13^ This pain is due to the irritation of sensory nerve fibres in the neurovascular bundles of marrow.^14^ An increase or change in the mechanical stress to bone, as was observed in this case, can result in bone marrow oedema and pain.

To date, the only reported successful treatment of a symptomatic “herniation pit” or “juxta-articular cystic lesion” has been surgical curettage.^8^ Regardless of the cause of these lesions we feel that image-guided steroid injection should be considered for such symptomatic lesions, especially if on initial imaging there is perilesional bone oedema as was seen in this case.

In summary, this report reviews the causes, imaging appearances and potential treatment of symptomatic herniation pits, emphasizing the role of radiologically guided intra-articular steroid injection.

## Learning points

1. Herniation pits or “Pitt’s pits” are small, well-delineated, lucent oval or sometimes lobulated and predominantly incidental lesions, found within the proximal superior quadrant of the femoral neck.
2. Originally they were felt to result from mechanical forces causing herniation of soft tissue and synovium through a defect in the bone. However, more recently an association with femeroacetabular impingement has been suggested as a possible aetiology.
3. Image-guided steroid injection should be considered for symptomatic lesions, especially if on initial MRI there is perilesional bone oedema following trauma as was seen in this case.

All procedures performed in studies involving human participants were in accordance with the ethical standards of the institutional and/or national research committee and with the 1964 Helsinki declaration and its later amendments or comparable ethical standards.

## Consent

Written informed consent for the case to be published (including images, case history and data) was obtained from the patient(s) for publication of this case report, including accompanying images.
